# “Some like it hot”: spectators who score high on the personality trait openness enjoy the excitement of hearing dancers breathing without music

**DOI:** 10.3389/fnhum.2014.00718

**Published:** 2014-09-11

**Authors:** Corinne Jola, Frank E. Pollick, Beatriz Calvo-Merino

**Affiliations:** ^1^Division of Psychology, University of Abertay DundeeDundee, UK; ^2^School of Psychology, University of GlasgowGlasgow, UK; ^3^Department of Psychology, City University LondonLondon, UK; ^4^Department of Psychology, Universidad Complutense de MadridMadrid, Spain

**Keywords:** action observation, personality, music, esthetic appreciation, entrainment

## Abstract

Music is an integral part of dance. Over the last 10 years, however, dance stimuli (without music) have been repeatedly used to study action observation processes, increasing our understanding of the influence of observer’s physical abilities on action perception. Moreover, beyond trained skills and empathy traits, very little has been investigated on how other observer or spectators’ properties modulate action observation and action preference. Since strong correlations have been shown between music and personality traits, here we aim to investigate how personality traits shape the appreciation of dance when this is presented with three different music/sounds. Therefore, we investigated the relationship between personality traits and the subjective esthetic experience of 52 spectators watching a 24 min lasting contemporary dance performance projected on a big screen containing three movement phrases performed to three different sound scores: classical music (i.e., Bach), an electronic sound-score, and a section without music but where the breathing of the performers was audible. We found that first, spectators rated the experience of watching dance without music significantly different from with music. Second, we found that the higher spectators scored on the Big Five personality factor openness, the more they liked the no-music section. Third, spectators’ physical experience with dance was not linked to their appreciation but was significantly related to high average extravert scores. For the first time, we showed that spectators’ reported entrainment to watching dance movements without music is strongly related to their personality and thus may need to be considered when using dance as a means to investigate action observation processes and esthetic preferences.

## Introduction

Dance is a multisensory performative art form that combines a number of elements, so-called strands. While the core strand of dance is movement, music is a fundamental companion. In fact, the genre of music defines the choreographic style (Jordan, 2000) and sets audiences expectations (Reason and Reynolds, 2010). For example, a classical ballet movement performed to hip hop music would not be considered a part of a classical ballet performance. Hence, the multisensory integration of movement and sounds in a performance are necessarily relevant for the complete audience’s experience. Most neuroscientific studies on dance have mainly focussed on just the visual aspect of movement. Therefore research combining movement and music is timely.

Research on the effect of music on movement in general, suggests that the coupling of music and movement is a “natural” process, which enhances the perception of emotion (e.g., Sievers et al., 2012). Neuroscientific research has shown evidence for shared neuronal substrates for music and movement perception (e.g., Janata and Grafton, 2003; Zatorre et al., 2007). Schmitz et al. (2013) further found that congruent compared to incongruent sonification of passively observed movements increased the activity not only in brain areas processing audio-visual integration, but also in wider a network of brain regions (e.g., frontal, inferior parietal and superior temporal) previously found to be activated during observation of familiar actions (e.g., Cross et al., 2009b). It is also well known that the integration of sound and action is not always mutually balanced. For example, visual information (perception of a movement/speech) can change the experience of the auditory stimulus, such as in the familiar McGurk effect (McGurk and MacDonald, 1976). Notably, for non-speech related auditory stimuli it is more common that audition affects vision than vice versa (Van den Stock et al., 2009; for a review see Shams and Kim, 2010). Given the research mentioned above, it is expected that dance perception can be highly modified by the properties of the accompanying music. One may for example predict that music has the ability to modify the endogenous perception of the rhythm of movements and thus change considerably the internal synchronization of audience members with the performers.

Studies investigating movement observation in general and dance perception in particular (without music), have followed an ideomotor approach, whereby observers perceive and simulate others’ actions based on their own action repertoire (see Knoblich and Sebanz, 2008). Dance observers’ brain activity is dependent on their previous physical experience with the observed movements (Calvo-Merino et al., 2005, 2006; Cross et al., 2006, 2009a; Aglioti et al., 2008; Orgs et al., 2008). Moreover, observers’ physical ability to perform a movement also modulates their esthetic appreciation for those particular movements (Cross et al., 2011). These as well as dance un-related studies (e.g., Braadbaart et al., 2014) suggest a close connection between the sensorimotor experience of the observer, its internal movement simulation or emotion expression, and its perceived physical and esthetic qualities (e.g., Cross et al., 2011). However, the co-occurrence effect of music or level of internal entrainment during dance observation still needs further discussion.

Factors that point towards underlying entrainment contingency in dance spectators were described in regards to the appreciation of dance. These are embodied verticality of dance stimuli (Calvo-Merino et al., 2008; Daparti et al., 2009) relevant for perception of entrainments’ well-known main factor rhythm (Cummins, 2009), as well as the physical co-presence of performers (Jola and Grosbras, 2013), which provide the context for successful social entrainment (e.g., Chartrand and Bargh, 1999). On the understanding that dance spectators’ motor simulation may be responsive to motoric entrainment of auditory and/or visual stimulation, then two important factors should receive further attention: How spectators’ responses are modulated by (1) music and/or sound (i.e., natural dance companions); and (2) spectators’ individual characteristics beyond physical or visual expertise (i.e., personality traits).

While some dance studies in neuroscience integrated a musical component (Brown et al., 2006; Cross et al., 2009b; Jola et al., 2012; Jola and Grosbras, 2013), the focus was not on the effect different types of music has on the perception of movements (for a review see Bläsing et al., 2012). Evidence for music-modified action observation was however shown in two recent studies. First, Jola et al. (2013) found increased synchronized brain activity in visual and multisensory areas across 11 participants when watching a classical Indian dance performance with music compared to when watching the same performance without related auditory stimulation. This study clearly showed that even early visual processing is modified by the musical component. Yet, the data did not allow specifying which factors in the combination of music and movement led to spectators’ increased synchronized entrainment. Second, Grosbras et al. (2012) found that participants’ subjective enjoyment ratings were attributed more strongly to the synchronicity and fluidity of music and movement after disruption of the parietal cortex by means of rTMS. The authors interpret their results in line with a “freeing up” of emotional processing by inhibiting the parieto-frontal executive control system (to which they account action observation processes). Importantly, the differences were only significant in the short section where the movements were accompanied by classical music. Hence, it remains unclear whether the enhanced relevance of music is due to a change in the music style or in the emotional response *per se*. Nevertheless, the study by Grosbras et al. (2012) indicates that participants’ personality in dealing with emotional processing could act as an underlying factor in the appreciation dance performed to music.

Music in itself has repeatedly been shown to affect listeners’ emotional and neural responses (for a thorough review see Juslin and Sloboda, 2011) dependent on their personality (Park et al., 2013). Of the Big Five personality factors (Costa and McCrae, 1992), Openness (i.e., extent of interest in new experiences) was repeatedly found to correlate with a variety of music style preferences, such as classical music, jazz, and soul whereas the excitement-seeking facet of Extraversion (i.e., extent of orientation towards the external world) was found to correlate with preference for rock music (for a review, see Rentfrow and Mcdonald, 2011). Further, behavioral evidence for a relationship between Neuroticism (i.e., extent of emotional instability) and classical music preference was reported (Dunn et al., 2012).

Meanwhile, effects of individuals’ personality dispositions on action observation processes and/or action understanding have predominantly focused on empathic ability. Strong evidence in support of a positive correlation between empathic abilities and enhanced internal sensorimotor resonance as part of the mirror neuron system ranges from visual observation of simple finger actions (Lepage et al., 2010), to observation of pain inflictions (Minio-Paluello et al., 2009), or watching complex dance movements (Jola et al., 2012). Moreover, purely listening to the sound of actions also activated the mirror neuron system more strongly for individuals with high empathic abilities (Gazzola et al., 2006). Yet no study has tested the effects of spectators’ personality traits on complex action observation neither with nor without accompanied sound.

We set out to explore the effects of spectators’ personality on their appreciation of specific combinations of music and movement. While the movement sequence was identical across conditions, the accompanied music was according to Rentfrow et al. (2011) reflective and complex (classical music), energetic and rhythmic (electronic sound-score), or without music, where the performers’ breathing was audible. Our rational was to access the effect of internal entrainment to the auditory stimuli on their watching dance experience. We predicted that auditory stimuli affect the perception of movement and that personality affects how these movements are evaluated and thus experienced. We thus expected personality dependent differences in the reported enjoyment of watching dance when the same movements are combined with different types of music. Based on previous findings on music and personality traits (e.g., Rentfrow et al., 2011; Dunn et al., 2012), if the music dominates the perception of dance, one would assume that spectators who score high on the personality factor Openness or Neuroticism would enjoy the dance movements accompanied to classical music, whereas spectators who score high on Extraversion and Agreeableness would enjoy watching the movements performed to the electronic sound-score. Further, according to known effects of personality abilities (i.e., expertise) on spectators’ motor resonance to watching dance, we also controlled for influences of visual and physical experience. In order to explore the links between spectators’ internal entrainment with dance movements performed to different types of music and their personality, we used the standardized, reliable Big Five Questionnaire (Neuroticism-Extraversion-Openness Five-Factor Inventory (NEO-FFI)). To our knowledge, no study has yet measured how audiences’ engagement with the dance performance is modulated by their personality.

## Method

### Participants

We collected responses in three separate experimental groups with psychology staff and students either from the University of Glasgow or the Open University. We did not have any restrictions on participation and grouped participants according to their dancing experience *post hoc*. The study was approved by the Ethics Committee of the Faculty of Information and Mathematical Sciences, University of Glasgow. All subjects gave their written informed consent prior to inclusion in the study. A total of 52 participants (37 female) with an average age of 36 years ± 1 (SD) watched a 24 min lasting dance performance in one of three experimental groups on a large video screen once. One group of 14 participants watched the original version of the dance performance with the sound scores in the order classical, no-music, electro. The edited version electro/classical/no-music was shown to a group of 21 participants and the order no-music/electro/classical to a group of 17 participants. Importantly, in all three groups, the presentation consisted of one uninterrupted continuous video.

Neither the number of males nor females differed significantly across the three presentation groups: Chi-square, df_(2)_ = 2.324, *p* = 0.313 for 8, 15, and 14 females, and df_(2)_ = 1.200, *p* = 0.549 for 6, 6, and 3 males, respectively. Between-subjects univariate ANOVA showed no significant age difference between the three groups, *F*_(2,49)_ = 1.373, *p* = 2.263. Furthermore, the overall liking ratings did not differ across the three condition orders, *F*_(2,49)_ = 0.154, *p* = 0.858.

The majority of the participants (72.9%) had a University degree (MA or PhD), 20.8% had a postgraduate degree or further education and for only 6.25% the most recent education was secondary school (education information was missing for four subjects). While few participants had some physical or visual experience in dance (see below), none of them were professional dancers.

Of all participants, two did not hand back the personality questionnaire and one participant did not provide information on visual experience. We thus had a remaining 49 participants. Of these, 25 participants had some physical experience in a variety of recreational dance styles and 24 indicated to have never taken any dance classes. In both of these groups, 10 participants had no previous experience in watching dance performances. We thus have a between-subjects factor “experience” with four groups: Physical and visual experience (*N* = 15, *“experienced”*), physical but no visual experience (*N* = 10, *“physical”*), visual but no physical experience (*N* = 14, *“visual”*), neither physical nor visual experience (*N* = 10, *“none”*).

### Measuring instruments and procedure

#### Dance video

Participants were asked to watch the dance piece “Double Points: 3×”, choreographed a dance piece by Rosie Kay, on a big screen. The piece was performed by the choreographer and a male dancer, Morgan Cloud and was a modification of an earlier version created in cooperation with the researchers of the Watching Dance Project^1^ to allow measuring the effect of music on movement perception. It was recorded in Manchester during a specifically staged life performance.

Double Points: 3× consists of an identical movement phrase repeated three times to different soundscapes danced between a short prelude and an epilog. The soundscapes that accompanied the movement phrases were Bach’s “Concerto for Oboe and Violin in C Minor, Allegro (condition “classical”), breathing (condition “no-music”, where no sound other than produced by the performers was audible), and an electronic sound-score composed by Ian Wallman (condition “electro”). The dance performance can be viewed in full length on http://paco.psy.gla.ac.uk/watchingdance. As can be seen in the video, the recording of the performance was a stable front recording capturing the whole stage with a Sony XDCAM PMW-EX1 in NTSC High Quality format of 1280 × 720 with 59.94 progressive frames per second. This setting was chosen to capture quick movements in best quality and the stable front recording allows the spectator to select their object of attention as in a life performance.

The digital recording of the live performance was then edited in order to balance the succession of the sound conditions across three spectator groups. The editing was possible without noticeable disruption, since the start and end body positions of a section were identical. The length of each section lasted 6 min and due to its length and complexity, spectators did not immediately recognize that the same movement phrase was repeated three times within the full performance.

#### Testing likeness ratings

All participants watched the video recording of the performance collectively as part of one of three groups in a dedicated darkened auditorium, projected on a screen of at least 177 × 175 cm, from a distance of at least 3 meters. After the screening of the full performance, participants were handed out a questionnaire including items on personal preferences, demographics, and motivations to watch dance. Participants scored how much they liked watching the performance on a 5 point Likert scale ranging from 1 for “strongly dislike”, to 2 for “dislike”, to 3 for “neither like nor dislike”, to 4 for “like”, and to 5 “strongly like” as a whole, as well as how much they liked watching the individual movement parts, i.e., the movement phrase performed to classical music, without music, and to an electronic sound-score. For each question, participants were asked to describe their experience in a few words.

#### Personality questionnaire

Spectators’ personality characteristics were measured by means of the “NEO-FFI”, a questionnaire that assesses the “Big Five” personality dimensions, which have been found to be measurable and reliable (e.g., McCrae and Costa, 1999; see also Rentfrow and Mcdonald, 2011). The NEO-FFI is a short version of the original Big Five questionnaire. It consists of 60 reliable items with a reported Cronbach’s α of between 0.71–0.85, (Borkenau and Ostendorf, 1993). It is frequently used in order to assess esthetic preferences (e.g., Chamorro-Premuzic et al., 2009; Rentfrow and Mcdonald, 2011), and its validity and dimensionality has been confirmed several times (Costa and McCrae, 1992).

The five “core” dimensions of the NEO-FFI are *Neuroticism* (e.g., “I often feel inferior to others”), *Extraversion* (e.g., “I really enjoy talking to people”), *Openness to experience* (e.g., “Sometimes when I am reading poetry or looking at a work of art, I fell a chill or wave of excitement”), *Agreeableness* (e.g., “I would rather cooperate with others than compete with them”), and *Conscientiousness* (e.g., “I pay my debts promptly and in full”). A short but comprehensive description of the five personality factors can be found in Weibel et al. (2010).

The NEO-FFI was handed out together with the other questionnaires after audiences watched the full performance on screen. The questions were answered on a 5-point Likert scale from strongly disagree (0) to strongly agree (4). The reliability (cronbach’s alpha) of the Big Five factors each consisting of 12 items in our tested sample (*N* = 49) was 0.89 for Neuroticism, 0.80 for Extraversion, 0.66 for Openness, 0.51 for Agreeableness, and 0.80 for Conscientiousness.

### Analysis

Since Likert scale data are essentially ordinal, we first inspected the liking ratings in regards to the frequency distribution of the individual scales. According to a recent critical review by Norman (2010), parametric statistics are however robust with respect to some violations, such as the use of parametric analysis on Likert scale data. In order to identify potential general rating tendencies of individual expertise groups, we thus analyzed the liking ratings based on the mean values across participants within conditions by means of SPSS Version 21.

We further tested for statistically significant correlations between the three combinations personality factors, ability, and liking ratings. In order to compare the relative appreciation of the three sound conditions Bach, electro, and no-music all spectators, we based our correlation analyses on the liking ratings of the three different sound conditions (classical, no-music, electro) on within-subjects standardized scores. We thus calculated individual subjects’ liking scores based on their mean and standard deviations, i.e., *y* = (*x*-mean_individual_)/SD_individual._ Such ipsatisation prevents acquiescent rating and further controls for extremes (Fisher and Milfont, 2010). As we were interested in the relative appreciation and since we found no effect of expertise on the means of the raw data in the analyses indicated above, the limitations of the interpretation on such standardized scores (dependency of rating in one condition compared to the other) are marginal in our study.

Only three subjects rated all sound conditions equally; hence, they received a score of zero for all three conditions. Since participants’ ipsatisation scores deviated significantly from a normal distribution for all conditions (df_(49)_ = 0.85, *p* < 0.001 (classical), df_(49)_ = 0.90, *p* < 0.001 (no-music), and df_(49)_ = 0.91, *p* ≤ 0.001 (electro), Shapiro-Wilk test), we decided to use a conservative approach of non-parametric tests (Spearman Correlation), also including *F*-statistics on ranked variables (Conover and Iman, 1982).

## Results

### Liking ratings

**Figure 1:**
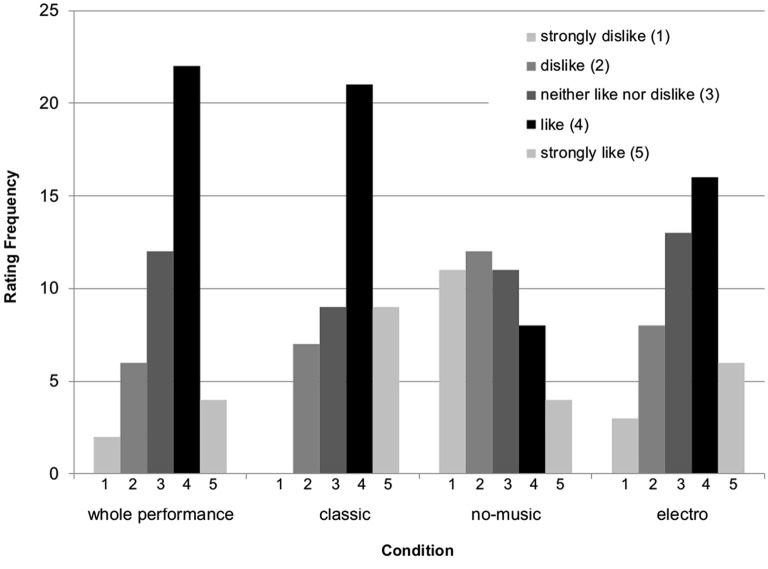
**Frequency distribution of the ratings from 1 (strongly dislike) to 5 (strongly like) for the performance as a whole and the three movement sections performed to classical music, no-music, and electronic sound-score**.

The average liking rating for the performance as a whole across all participants was 3.43 (±1.00 SD) on a scale from 1 (strongly dislike) to 5 (strongly like). On average, the dance movements performed to classical music received with 3.69 (±0.98 SD) the highest rating. The no-music condition was rated lowest with 2.67 (±1.30 SD), whereas the electro was rated 3.33 (±1.13 SD). It is important to note that whilst the no-music condition was indeed rated to be liked the least on average, the distribution of the ratings was large (Figure 1). Non-parametric chi-square tests confirmed that the frequency distribution of the likeness ratings are significantly deviating from an equal distribution in all but the no-music condition: Chi-square = 10.510, df = 3, *p* = 0.015 (classical); 3.755, df = 4, *p* = 0.440 (no-music); 11.918, df = 4, *p* = 0.018 (electro).

### Expertise and liking

**Figure 2:**
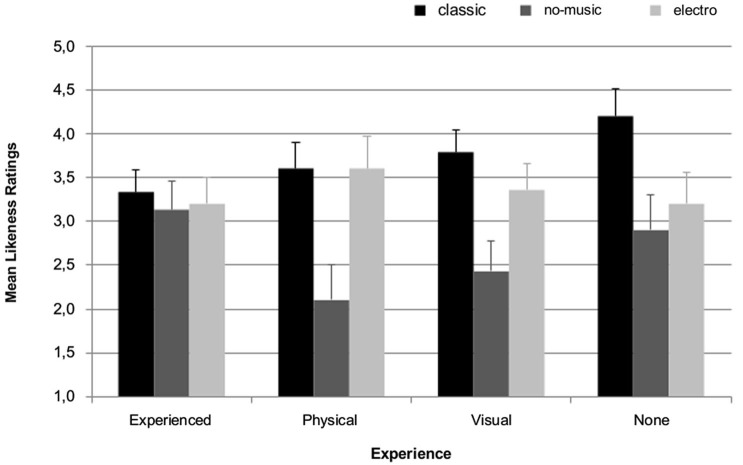
**Mean and standard error likeness ratings (*N* = 49) from 1 (strongly dislike) to 5 (strongly like) for each sound condition (classic, no-music, electro) dependent on the type of expertise: experienced (physical and visual experience), physical (physical but no visual expertise), visual (visual but no physical experience), none (neither physical nor visual experience)**.

As can be seen in Figure 2, the ratings differ in respect to experience: participants who have both physical and visual experience show a general tendency to rate the performances at medium level, with similar scores for all three sound conditions. As a consequence, the no-music condition is rated higher in the experienced group compared to all other groups. However, tests of repeated measures ANOVA for the factors sound (classic, no-music, electro) and experience do not show a significant interaction between the two factors, *F*_(6,90)_ = 1.819, *p* = 0.113. While the main factor sound shows a significant effect, *F*_(2,90)_ = 13.244, *p* ≤ 0.001, the between-subjects factor experience does not, *F*_(3,45)_ = 0.357, *p* = 0.784.

Bonferroni corrected paired samples *t*-tests between the three conditions showed that the dance section in the no-music condition was liked significantly less than when accompanied by either the classical or the electronic music, *t*_(48)_ = 4.168, *p* < 0.001, and *t*_(48)_ = 3.433, *p* = 0.004, respectively. The ratings are not significantly different between the classical and the electro condition, *t*_(48)_ = 4.168, *p* = 0.258. The same effects are found for ipsatisations scores which are used in the subsequent covariance and correlation analyses.

### Personality and liking

**Figure 3:**
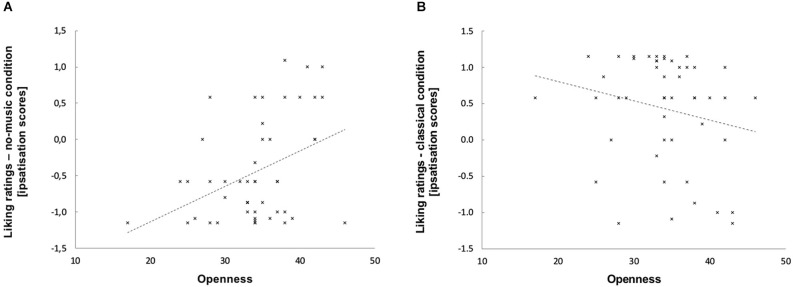
**(A) (left) and (B) (right)**. Correlation and linear trend between the scores on the personality factor Openness (*x*-axis) and the standardized liking ratings for the breathing condition (*N* = 49; *y*-axis).

Repeated measures ANCOVA for the grouping factor sound (classic, no-music, electro) on the ranked dependent variables liking and the personality traits covariances (Neuroticism, Extraversion, Openness, Agreeableness, and Conscientiousness) showed a significant main effect for the factor sound, *F*_(2,86)_ = 4.606, *p* = 0.013. The covariance with the personality trait Openness was significant, *F*_(2,86)_ = 6.008, *p* = 0.004. The personality trait Conscientiousness showed a strong trend to act as a covariance on sound, *F*_(2,86)_ = 2.872, *p* = 0.062. All other personality traits revealed significance values >1.000. Bilateral non-parametrical spearman’s correlation for the personality factors Openness and Conscientiousness and the standardized liking scores (classical, no-music, electro) showed no significant correlation with Conscientiousness (significance values >1.100) but two relevant correlations with the factor Openness. First, we found a significant positive correlation for the liking scores of the no-music condition with Openness: the higher spectators scored on Openness, the more they liked the no-music condition, *r* = 0.377, *p* = 0.008 (see Figure 3A). Second, a trend for a negative correlation between Openness and liking ratings could be observed for the classical condition: Spectators who gave higher liking ratings for watching the dance movements accompanied by classical music scored lower on the personality factor Openness, *r* = −0.242, *p* = 0.093 (see Figure 3B).

### Personality and experience

**Figure 4:**
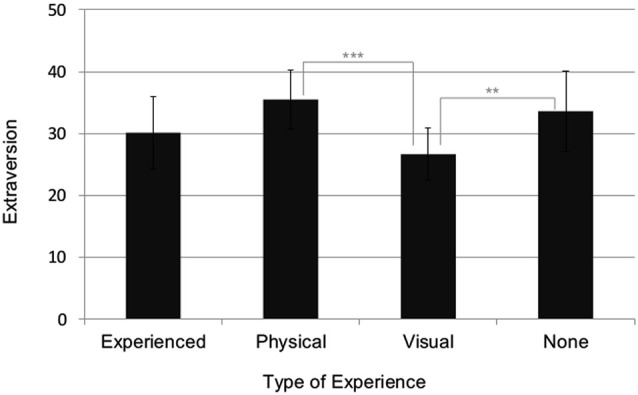
**Differences (Mean and SD) in the Extraversion personality scores (*y*-axis) for the different experience groups (*x*-axis)**. *** = *p* ≤ 0.001, ** = *p* ≤ 0.005.

Non-parametric analysis on the link between experience and personality showed that only the personality factor Extraversion is significantly related to experience, Chi-Square, df_(3)_ = 15.05, *p* = 0.002 (Kruskal Wallis Test), but none of the other personality factors, df_(3)_ = 5.43, *p* = 0.143 (Neuroticism), df_(3)_ = 3.37, *p* = 0.339 (Openness), df_(3)_ = 4.60, *p* = 0.204 (Agreeableness), df_(3)_ = 2.32, *p* = 0.509 (Conscientiousness). As indicated in Figure 4, independent non-parametric Mann-Whitney tests showed that spectators who have only visual experience scored on average significantly lower on the personality factor Extraversion than the group who had either only physical or no experience at all, *U* = 6.50, *p* < 0.001, and *U* = 22.50, *p* = 0.005, respectively. Extraversion did not differ between those who had no experience and those who were experienced:* U* = 59.00, *p* < 0.373; or between those who had no experience and those with physical experience: *U* = 40.00, *p* = 0.446. The Extraversion scores showed a trend to be lower for those with only visual experience than for those who had both visual and physical experience, *U* = 65.00, *p* < 0.080. Furthermore, those with physical expertise showed a strong trend for higher Extraversion scores than those with both visual and physical expertise,* U* = 42.00, *p* < 0.065.

## Discussion

We investigated the effect of three types of audio stimulation on spectators’ entrainment with dance movements presented within a continuous dance performance. The effect was studied in consideration of spectators’ personality (i.e., in form of the Big Five personality traits) and their ability (i.e., in form of visual and/or physical dance experience). Spectators were asked to rate on a 5-point Likert scale how much they liked the dance movements performed to classical music, performed without music but audible breathing, and performed to an electronic sound-score. Given previous research on personality and musical preferences, we expected spectators’ appreciation of watching dance to be modulated by their personality scores. In particular, if music is dominating the perception of watching dance moves, spectators with high scores on Openness or Neuroticism were expected to enjoy it more when classical music accompanied the movements; and spectators with high scores on Extraversion or Agreeableness to enjoy it more when an electronic sound-score was played. In line with previous research, we also explored whether physical and/or visual experience enhances motor entrainment and esthetic appreciation.

Overall, we found evidence that watching dance accompanied by different types of audio scores significantly modifies spectators’ experience. Our audiences showed a general preference for the dance movements when accompanied by classical music. When the identical movement sequence was performed by the very same performers to an electronic sound-score or without music, the average enjoyment ratings across all participants were significantly lower. One could therefore suggest that the particular types of movements performed to Bach’s Concerto for Oboe and Violin in C Minor built a coherent whole that was most popular across our audience. The idea that there is a hierarchy of musical styles with classical music being at the top is prevalent in western-industrialized countries (Martin, 1995). It must be therefore considered that the preference ratings found here may be specific for our UK-based sample. Notably, however, while several musical pieces of Bach were created specifically for dance and his music has been present over a long period in the history of dance (Little and Jenne, 2002), the use of classical music in contemporary dance as shown here, is unusual.

Yet, beyond the effect of music, our spectators’ appreciation of watching dance movements did not reveal strong links between either their experience and enjoyment or their personality and music (i.e., classical or electronic music). In fact, the personality factor Openness showed a trend for a negative correlation with how much spectators liked watching the movements performed to classical music. This negative correlation may be explained by the unconventional pairing of classical music with contemporary dance. Moreover, some spectators who highly enjoyed the movements performed to classical music emphasized the enjoyment over the type of music above movement indicating a more conventional (i.e., less open to new experiences) rating style.

Notably, however, the condition with no music, where performers’ breathing was audible, showed two relevant significant effects. First, watching dance performed live without music (no-music condition) led to *qualitatively* distinct audience experience. In this condition, the variability of the ratings was highest, and an equal distribution of the frequency of the Likert scale ratings across spectators could not be rejected. Second, the valence of the audience experience to hearing the performers’ breathing is significantly positive if spectators score high on the personality trait Openness (i.e., the higher spectators scored high on Openness, the more they appreciated the no-music condition).

As Reason and Reynolds (2010) suggest, differences in tastes can be expected, but what is relevant for the no-music condition is that distinct interpretative strategies that can be identified. Reason and Reynolds (2010) conducted a qualitative audience research on their experiences based on several live performances, including Rosie Kay’s “Double Points: 3×”. The authors report that about half of an audience enjoy hearing the dancers’ respiration, as it brings performers and spectators viscerally closer, which increases intimacy. These spectators engage with a dance performance that provides them with a sense of rawness, closeness, and awareness of physical intensity and intimacy, and have thus been referred to by Reason and Reynolds (2010) as “sensualists”. The other audience members, for whom the audible respiration was uncomfortable and too intimate, were described as “escapists”, seeking a suspension from reality.

Our spectators’ subjective descriptions provided in addition to their Likert-scale judgments showed a similar disparity between their experiences: They either expressed strong negative or positive attributions to the thrill and excitement they experienced when sensing the performers’ physicality through the audible breathing. Spectators who disliked the no-music condition state that they found it “disturbing”, “eerie” and that it made them feel “anxious”, “uncomfortable”, “awkward”. Spectators who liked to hear the breathing stated it was “fabulous”, “impressive”, and they “enjoyed the enhanced sensory experience”. We describe this differential experience with the metaphor “some like it hot”, which is a widely used expression to describe the positive response of some audience members who enjoy the thrill and excitement of a specific emotionally charged genre of jazz music whereas another group of audience members do not enjoy their responses to a performance that is too lively (i.e., visceral).

The peculiar response to the no-music condition with audible respiration has potentially huge theoretical impact on the interpretation of existing and future research as most studies on spectators’ responses to watching dance are based on action observation without music or sound. In fact, it has previously been shown that hearing the sound of motor actions is sufficient to trigger motor resonance of the actions in the passive listener (e.g., Gazzola et al., 2006). However, while action sounds were found to evoke mirror neuron activity, sounds of biological motion, such as hearing other peoples’ footsteps, did not (e.g., Saarela and Hari, 2008). According to the hierarchical model of social interaction (see Knoblich and Sebanz, 2008), it is thus possible that performers’ respiration may not affect spectators on the level of motor resonance (proposed to serve action understanding), but on the lower level of social triggered physiological entrainment (proposed to affect emotional response). To our knowledge, there are yet however no empirical investigations on how physiological co-regulation between performer and spectator may affect spectators’ experience in passive action observation. Notably though, spectators descriptive explanations indicate strong emotional responses, supporting the idea of two forms of spectators’ resonances linked to the degree to which they are open to new experiences.

The personality factor Openness measures the extent to which somebody is prepared to expose themselves to new sensory experiences and further underlies spectators’ evaluation of their experience of intensity and intimacy created by the audible breathing. Moreover, individuals with high scores on Openness have an increased tendency to fantasize and daydream, notably with the aim to intensify their experiences. Spectators who scored high on the fantasy subscale of the emotional quotient have previously been found to show increased motor resonance when watching Indian dance with music (Jola et al., 2012). Based on these findings and the understanding of the emotional benefits of cross-person physiological coupling (see Helm et al., 2012), it is possible that sensualist spectators’ are more open to synchronize their respiration with the performer leading to enhanced action observation and alternated interpretations of the experience likewise.

An alternative explanation of our findings is that the no-music condition was perceived as more novel than the music conditions and thus exciting. Individuals with high scores on Openness do indeed prefer novelty and variety over familiarity and routine. Although few spectators mentioned the unconvential combination of movements with breathing, our design and results do not fully support an effect of familiarity/novelty. Importantly, the sound conditions were counterbalanced and thus the combination of movement and music/no music conditions were seen in different order of appearance. Notably, we found no order effect as it would be expected if familiarity or novelty during the experimental procedure would play a role. Moreover, while “Openness personalities” have a deep appreciation for art and beauty, are moved by poetry, and intrigued by art, importantly, one characteristic of Openness is that they are often found to be absorbed in music. We therefore propose that hearing the performers’ breathing divides spectators’ appreciation in two groups, those “who like it hot”, and those who don’t. In other words, the positive or negative valence spectators give to the sensation of experiencing the excitement and thrill through the performers’ breathing (through internal entrainment), dictates their appreciation and potentially their motor resonance. For example, Nummenmaa et al. (2014) found that sharing a sensation enhances neuronal synchronization. Hence, if spectators enjoy the viscerality that hearing the breathing evokes, then they could potentially show higher synchronization. A very recent study by Launay et al. (2014) showed that synchronization to the sound of a partner increases their likability. However, additional studies that compare neuronal processes between audience members in response to music and sound are needed to further clarify these suggestions.

While our findings open new avenues to discuss how individual differences affect spectators’ entrainment and motor resonance mechanism, one crucial limitation is that we have no direct evidence of spectators’ entrainment. Measuring neuronal or physiological coupling between performers and spectators is a difficult undertaking. A number of studies showed evidence in support of links between motor resonance and personality traits. First, motor cortex excitability (which is related to “entrainment” in action observation processes) was found to be correlated with personality traits (anxiety-related personality; Wassermann et al., 2001). Second, empathic abilities, such as the ability to fantasize (Jola et al., 2012) or the ability to empathize (Singer et al., 2004) were found to increase mirroring resonance in passive spectators. Finally, since motor cortices were found to be increased in response to volitional respiration (Evans et al., 1999; McKay et al., 2003) respiration rate may be considered an additional measure of interest in action observation studies. Finally, direct measures of entrainment should allow testing whether entrainment is a necessity for esthetic appreciation of dance.

A further limitation of our study is related to the measurement scale. Correlation analyses on the basis of 5-point Likert scales are not adequately discriminative. We thus transformed the ordinal data into ipsatisation scores, which make the conditions notably dependent from one another. While we believe that the inter-dependence of the standardized scores require consideration in the interpretation of the data, we do not assume that participants—as they saw one whole performance—rated the conditions independently, with the potential exception of the most experienced group. Although not significantly different, the ratings of the participants with both visual and physical experience rated the three identical movement sequences as more equally pleasing, showing a more expert-holistic rating of the performance as a whole, independent of the accompanying sound/music—whereas the other groups contrasted the three conditions with each other. The relationship between holistic perception and expertise has been already suggested in other studies using dance experts (Calvo-Merino et al., 2010; Stevens et al., 2010). Overall, these data suggest new venues to explore further relationships between types of processing, types of expertise (i.e., visual and physical, empathic abilities) as well as personality traits.

Notably, we found that spectators’ experience was significantly related to the personality factor Extraversion. Spectators’ who reported physical experience only, scored significantly higher on Extraversion than spectators who had visual experience. Individuals with high scores on Extraversion are active individuals who seek interactions with the environment and enjoy social groups. They do not mind to expose themselves to others. These are clearly all characteristics that are needed in dance practice. Based on the knowledge that dancers share genetic predispositions that are linked to personality types (Bachner-Melman et al., 2005) and that personality factors may influence action observation processes (e.g., Avenanti et al., 2009; Jola et al., 2012), a careful assessment of the heterogeneity of personality factors may be relevant when evaluating preference or similar process during action observation of dance, an in particular when physical experience plays a role.

One important aspect of our study is that the emotional content of our stimuli is ambiguous and thus open to audiences’ interpretation. Most studies on action observation are designed to elicit emotionally reactive empathy (e.g., viewing painful stimuli). For example, evidence for the relationship of entrainment and personality traits was shown in non-artistic actions (Liuzza et al., 2014). These authors found that motor resonance was selectively suppressed for immoral actions when viewed by participants who scored high on harm-avoiding personality traits. Moreover, Borgomaneri et al. (2013) found that positive and negative stimuli are not processed significantly differently at later time points, suggesting arousal being the relevant factor.

A limiting factor is that we tested spectators’ responses to previously recorded video. It is well known that personality and the experience of presence in virtual environment are related (Baños et al., 1999; Beeli et al., 2008; Weibel et al., 2010). Notably, the video presentation had considerable benefits. First, the conditions of the order could be controlled and second, the timing between performances adjusted. It has been shown that moving without music (i.e., when no musical cues are present) time lapses across performers are increased (Stevens et al., 2009). The video editing allowed us to adjust the duration of the performers’ inhaling to make it more comparable with the timing of the musical cues (none of these adjustments were not noted by spectators). Although we found similar audience responses as Reason and Reynolds in their qualitative study after the live performance, further studies should consider the audiences’ response to audible respiration between live and video, in particular since differences in motor resonance were found (e.g., Jola and Grosbras, 2013).

In contrast to music (e.g., Brattico and Jacobsen, 2009) and the visual arts (see Chamorro-Premuzic et al., 2009) relative little is known on individual characteristics that may affect the appreciation of dance and in particular if these are modified by specific combinations of movement and music. It is interesting to note that dance esthetics is a relatively recent field. Moving closer to other disciplines that look into the relationship between personality factors, internal entrainment with the performers’ movements and esthetic appreciation should be of future interest where synchronization in time or affect through movement is expected.

To summarize, for the first time, we showed empirical evidence that the accompanying sound or music has a strong effect on how otherwise identical dance movements are enjoyed. Based on the audiences’ verbal reports and our knowledge on motor entrainment to auditory and visual rhythmic stimuli, we propose that enhanced or reduced internal entrainment underlies the subjective ratings. We suggest that our understanding of spectators’ responses to watching dance may benefit from paradigms and interpretations dissociating entrainment and motor resonance, and considering how these processes may be also modulated by personality.

Spectators are aware that dance is intrinsically liked to music. The impact of our findings is thus twofold, with important theoretical and practical implications. A better understanding of personality effects on spectators’ internal entrainment with dance has a strong real-life applied potential by informing artistic management on how audiences’ can be targeted based on their personality for specific performance types. The knowledge on effects of personality should allow audiences to make more informed choices when buying tickets. Then, our finding also points towards the necessity of a careful re-evaluation of the theory of a general multisensory expertise-related motor resonance in action observation. This is in particular evident as most action observation studies presented actions in absence of sound.

Our study extends previous findings on the conditions that facilitate or inhibit motor simulation of spectators in response to complex natural, multi-sensory stimulation. A better understanding of these conditions will increase predictability of whether people will oppose or engage with other peoples’ actions, which is of huge relevance in planning successful social interactions. Future research investigating cognitive and neural responses related to watching dance, or associated processes, should therefore take into consideration the presentation or absence of music, and how spectators’ personality may modulate neuronal responses. Spectators may reveal different degrees of embodiment during dance and/or action observation dependent on their personality, which demands careful consideration of experimental group homogeneity in future studies.

## Conflict of interest statement

The authors declare that the research was conducted in the absence of any commercial or financial relationships that could be construed as a potential conflict of interest.

## Footnotes

^1^http://www.watchingdance.org
